# Secondary Glaucoma Resulting From Choroidal Melanoma in a Patient With Congenital Nevus of Ota

**DOI:** 10.1155/crop/1434134

**Published:** 2025-12-17

**Authors:** Sakaorat Petchyim, Supredee Pongrujikorn, Felix Paolo J. Lizarondo, Woraphong Manuskiatti

**Affiliations:** ^1^ Department of Ophthalmology, Faculty of Medicine Siriraj Hospital, Mahidol University, Bangkok, Thailand, mahidol.ac.th; ^2^ Department of Dermatology, Faculty of Medicine Siriraj Hospital, Mahidol University, Bangkok, Thailand, mahidol.ac.th

**Keywords:** acute angle closure, choroidal melanoma, nevus of Ota, secondary angle closure, uveal melanoma

## Abstract

**Purpose:**

First, to report nevus of Ota accompanying ocular melanoma in Thai patients. Second, to report angle‐closure glaucoma as a presentation secondary to ocular melanoma along with a pathological report giving insight into the pathophysiology of secondary glaucoma in uveal melanoma.

**Observations:**

This case describes a patient with a nevus of Ota who experienced gradual, painless vision loss in the left eye, coinciding with the nevus location. Over 7 months, the patient′s vision progressively worsened, culminating in the diagnosis of rhegmatogenous retinal detachment. Tumor identification was first achieved through fundus examination at a tertiary care center, and the tumor was subsequently confirmed via ocular ultrasonography. The patient elected against eye enucleation. The patient presented again as a secondary angle‐closure glaucoma resulting from ocular melanoma.

**Conclusions and Importance:**

This case highlighted the importance of a rare malignant tumor that can accompany a nevus of Ota. Patient symptoms can vary from visual loss to a painful eye. Physician must be aware of the disease and include melanoma in the differential diagnosis in patients with a nevus of Ota. Thorough eye examination is important. Ocular ultrasonography is feasible, simple, and crucial for diagnosis. Accurate staging is vital for choosing the correct treatment strategy to save the patient′s life.

**Summary**

• Malignant transformation of nevus of Ota is notably rare in Asian populations [1].
• Chronic retinal detachment in this patient led to a delayed diagnosis.
• Ocular ultrasonography is essential for early detection in such patients.
• Extensive tumor growth can cause secondary angle‐closure glaucoma.
• Clinical and pathological evidence supports various secondary glaucoma mechanisms in uveal melanoma.

## 1. Introduction

Nevus of Ota, or oculodermal melanocytosis, manifests as a dermal melanocytic hamartoma that typically presents as a bluish‐brown patch along the first and second branches of the trigeminal nerve [2]. The lesion is congenital. The condition tends to affect the Asian population, with a higher incidence in females [3, 4]. However, its malignant transformation into melanoma is more common in Caucasians [5]. Melanomas arising from nevus of Ota may present in ocular structures, the central nervous system, or on the skin [6, 7–9]. The clinical presentation of ocular melanoma associated with nevus of Ota varies markedly from asymptomatic to causing visual loss, depending on the size and location of the tumor [10, 11]. Secondary glaucoma is an infrequent but serious complication of ocular melanoma [12, 13].

As of this writing, there have been only five reported cases of nevus of Ota accompanying ocular melanoma in the Asian population [11]. This report details such a case identified in Thailand.

## 2. Case Report

### 2.1. Initial Presentation

A 66‐year‐old Thai woman was referred to the Ophthalmology Department at Siriraj Hospital for chronic rhegmatogenous retinal detachment in her left eye. She had noticed blurred vision 7 months prior to the detachment being diagnosed. She had cataract surgery for both eyes some years ago. The patient′s medical history was unremarkable, except for a significant smoking history of five packs of tobacco per year over a span of 30 years, totaling 150 packs.

At presentation, her visual acuity was limited to hand motions in the left eye and 6/9 in the right eye. An examination of the left eye revealed generalized scleral melanocytosis with dilated, tortuous vessels and hyperpigmented iris. Gonioscopy showed an open angle and hyperpigmented trabecular meshwork in the left eye, with no neovascularization or blood in Schlemm′s canal. The anterior chamber was clear, but the vitreous was slightly hazy, with 2+ cells. Both eyes were pseudophakic. Dilated fundus examination of the left eye unveiled a subretinal mass in the superior retina surrounded by subretinal hemorrhage and shallow retinal detachment. According to Vishnevskia et al. [14] proposed classification for ocular involvement in Nevus of Ota, this patient has at least 2D+ with lack of detail about choroidal melanosis due to retinal detachment obscuring the underlying choroid.

Additionally, the patient had a hyperpigmented skin lesion on the left side of her face, covering the upper and lower eyelids, forehead, and cheek (Tanino′s classification Type II) [15]. Systemic examination detected rhonchi in the left lung but no palpable lymph nodes or hepatomegaly.

**Figure 1:**
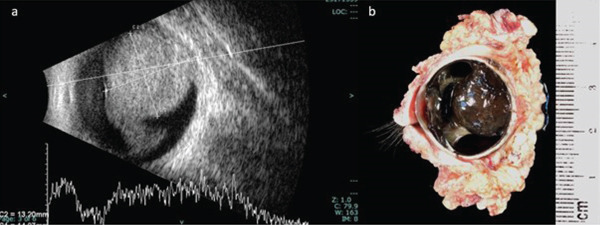
(a) Ocular sonography showing mass attached to choroid at the superior section of vitreous cavity. (b) Sagittal section of left eyeball with periorbital soft tissue, and both eyelids demonstrates an intraocular, dark tan mass.

Initial ocular ultrasonography (Figure 1a) revealed a mass measuring 13.95 by 14.01 mm located in the superior vitreous cavity and attached to the choroid. The mass exhibited medium internal echogenicity and lacked choroidal excavation suggesting a diagnosis of choroidal melanoma.

Subsequent diagnostic procedures encompassed magnetic resonance imaging of the brain and orbits, chest X‐ray, abdominal ultrasound, complete blood count, platelet count, blood urea nitrogen, creatinine, electrolytes, liver function tests, and tumor markers (CEA, CA 19–9, CA 125, and AFP). All the results were within normal limits, indicating no evidence of metastasis.

### 2.2. Nonocular Condition

The patient was referred to a dermatologist for an evaluation of the hyperpigmented lesion. Since the lesion had been present from birth, the dermatologist clinically diagnosed it as a nevus of Ota. Considering the choroidal mass and the suspected diagnosis of choroidal melanoma, an incisional biopsy was conducted. The biopsy confirmed dermal melanocytosis.

### 2.3. Secondary Glaucoma

An ophthalmologist recommended that the left eye be enucleated, but the patient declined the procedure and was lost to follow‐up. However, she returned 10 months later with severe pain in her left eye, which she had been experiencing for 2 days prior to seeking medical attention at her local hospital. There, she was diagnosed with neovascular glaucoma associated with nevus of Ota.

Her visual acuity had deteriorated to no light perception in the left eye and was 20/50 in the right eye. The intraocular pressure was 12 mmHg in the right eye and 43 mmHg in the left eye. The left eye showed severe chemosis, Grades 1–2 corneal haze, a shallow anterior chamber with 360° iridocorneal contact peripherally, two corneal thicknesses centrally, prominent pigmented cells in the anterior chamber, and a 4‐mm fixed pupil with a relative afferent pupillary defect. Computed tomography scans of the brain and orbits indicated anterior intraocular lens dislocation and an enlarged, enhancing left lacrimal gland. These findings did not rule out extraocular tumor invasion, leading to a recommendation for exenteration of the affected eye. Systemic evaluation for metastasis showed no distant spread except for chronic pulmonary embolism, which was deemed an incidental finding by internal medicine specialists.

### 2.4. Pathological Specimen

**Figure 2:**
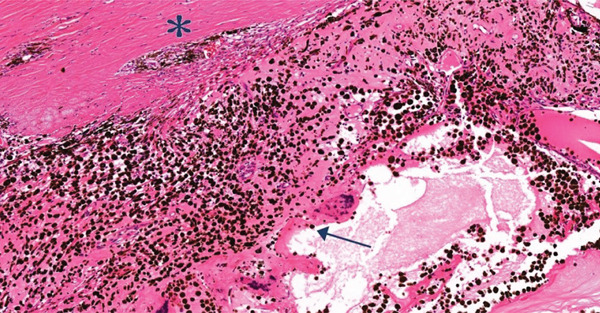
Melanoma epithelioid cell type at trabecular meshwork (asterisk) and ciliary body (arrow) (H&E ×10).

**Figure 3:**
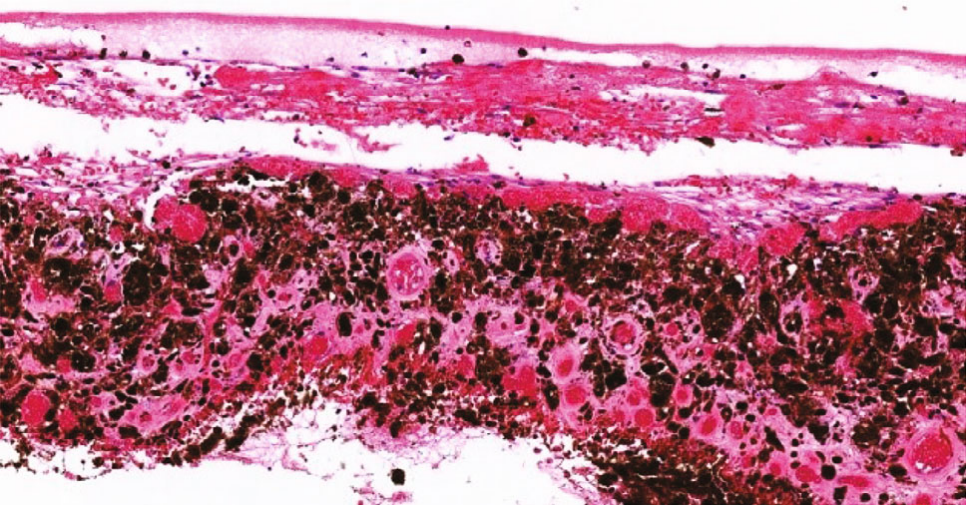
The iris shows neovascularization (H&E ×20).

A gross examination of the specimen (Figure 1b) revealed a solid, dark tan intraocular mass. It measured approximately 20 mm in base diameter and 15 mm in height at the cut edges, primarily involving the nasal aspect of the globe and extending from 6 mm from the optic nerve head to the limbus. Pathological examination confirmed choroidal melanoma of the epithelioid cell type. The tumor had penetrated the sclera without optic nerve head or vascular invasion. Based on the American Joint Committee on Cancer Eighth Edition, the tumor was classified as pT4d, indicating a Category 4 size with ciliary body involvement and extraocular extension under 5 mm in diameter. Notably, tumor cell infiltration of the ciliary body and trabecular meshwork (Figure 2) and iris neovascularization (Figure 3) were identified as potential contributing factors to the secondary glaucoma observed in this eye.

## 3. Discussion

This patient with nevus of Ota had a complex clinical course. Initially experiencing chronic blurred vision, he was diagnosed with chronic rhegmatogenous retinal detachment, a condition that unfortunately masked the underlying pathology.

A report from the United Kingdom indicates that in approximately 23% of uveal melanoma cases, the tumor is initially missed, leading to more advanced disease and an increased necessity for primary enucleation [16]. The clinical presentation of uveal melanoma varies based on tumor size and location, with symptoms ranging from asymptomatic to visual loss and eye pain [5, 17]. In our reported case, the patient experienced vision loss due to retinal detachment, a consequence of substantial subretinal tumor growth. This condition involved the macula extensively, leading to profound visual impairment and ultimately prompting the patient to seek medical advice.

Currently, there are no established guidelines for routine eye examinations in patients with nevus of Ota. Clinicians who may encounter these patients should remain vigilant about the potential for malignant transformation [11]. This is particularly important when patients present with abnormal symptoms such as visual loss, orbital pain, visual distortion, or proptosis. Recent ocular imaging such as fundus autofluorescence and enhanced depth spectral domain optical coherence tomography assist in the detection of early‐stage melanoma and in distinguishing melanoma from nevi.

A detailed medical history and thorough ocular examination are critical for diagnosis. In our reported case, a key finding in the anterior segment was scleral melanocytosis accompanied by dilated tortuous vessels, potentially indicative of a sentinel vessel often seen in ciliary body melanomas [18]. The posterior segment presented a classic appearance: a dome‐shaped, subretinal, pigmented mass [19]. Ocular ultrasonography, an affordable and effective diagnostic tool, helps ascertain tumor location, size, and any extrascleral extension [20]. According to the Collaborative Ocular Melanoma Study guidelines, the tumor found in our patient was classified as large, given that it exceeded 8 mm in thickness [21]. Given these circumstances, enucleation rather than eye‐conserving therapy is the preferred treatment option for our patient.

Shields et al. reported that each millimeter increase in tumor thickness, as determined by ultrasonography, is associated with a 5% greater risk of metastasis [22]. Given that the tumor in our patient measured 14 mm in thickness, there was a considerable risk for metastasis. Additionally, data from the Collaborative Ocular Melanoma Study indicate that at the time of tumor diagnosis, fewer than 4% of patients exhibit no signs of metastasis. This underscores the importance of conducting a metastasis evaluation early in the diagnostic process. Common metastasis sites are the liver, lungs, and bones [23]. In our reported case, the metastatic workup results were negative at both visits.

After initially declining enucleation, our patient later sought medical help for severe eye pain and elevated intraocular pressure.

Several mechanisms can account for the secondary glaucoma observed in our patient. The most likely mechanism of secondary glaucoma in this case is acute angle‐closure glaucoma due to the rapid development of eye pain within 2 days, along with a shallow anterior chamber, and a mid fixed dilated pupil. Another possible mechanism is neovascular glaucoma as evidenced by blood vessels on the iris, a finding confirmed by the pathological specimen (Figure 3).

Direct invasion by tumor cells is another possible mechanism. At the first visit, a hazy vitreous with cells suggested vitreous seeding, while at the second visit, large pigmented cells observed in the anterior chamber could have obstructed the trabecular meshwork. Pathological examination revealed direct angle invasion by the tumor (Figure 2). These secondary glaucoma mechanisms have been reported in previous literature with choroidal tumor both melanoma and metastasis [14, 24–27].

## 4. Conclusions

Our literature review conducted on January 15, 2024, using PubMed and Google Scholar with the keywords “uveal melanoma” and “Ota,” found no previous reports of uveal melanoma in Thai patients with Nevus of Ota. Physicians must be aware of the low incidence yet high risk of misdiagnosis, which can significantly impact patient morbidity and mortality. The clinical manifestations may vary from visual loss to secondary glaucoma. Thorough eye examinations and ocular ultrasonography are crucial diagnostic measures. Prompt systemic evaluations for distant metastasis are necessary. Accurate staging is vital for choosing the correct treatment strategy to save the patient′s life.

## Consent

Informed consent for publication of this case report was obtained. No personal identifying information is disclosed in this report.

## Disclosure

The authors did not receive support from any organization for the submitted work.

## Conflicts of Interest

The authors declare no conflicts of interest.

## Author Contributions

All the authors meet the International Committee of Medical Journal Editors (ICMJE) criteria and have significantly contributed to this manuscript. Sakaorat Petchyim: conceptualization, resources, writing—original draft, and writing—review and editing. Supredee Pongrujikorn: visualization. Felix Paolo J. Lizarondo: writing—review and editing. Woraphong Manuskiatti: conceptualization and supervision.

## Funding

No funding was received for this manuscript.

## Data Availability

The data that support the findings of this study are available on request from the corresponding author. The data are not publicly available due to privacy or ethical restrictions.
